# Workers’ Occupational Dust Exposure and Pulmonary Function Assessment: Cross-Sectional Study in China

**DOI:** 10.3390/ijerph191711065

**Published:** 2022-09-04

**Authors:** Wei He, Nan Jin, Huaxin Deng, Qi Zhao, Fang Yuan, Fengqiong Chen, Huadong Zhang, Xiaoni Zhong

**Affiliations:** 1 School of Public Health, Chongqing Medical University, Chongqing 400016, China; 2 Department of Occupational Health and Radiation Health, Chongqing Center for Disease Control and Prevention, Chongqing 400042, China

**Keywords:** occupational health, dust exposure, pulmonary function, pneumoconiosis

## Abstract

Objective: This study aims to determine the occupational health status of workers exposed to dust and the risk factors of lung function decline, to provide a basis for formulating corresponding occupational disease-prevention strategies. Methods: Data on 2045 workers exposed to dust, including their age, gender, exposure time, chest X-ray test results, and pulmonary function test results, were obtained from a key occupational disease monitoring project in Chongqing, China, in 2021. Chi-square tests and multifactorial logistic regression, and other methods, were used for statistical analysis. Results: The prevalence of pneumoconiosis-like changes was 0.83% (17/2045), and the prevalence of abnormal forced expiratory volume in one second (FEV_1_), forced vital capacity (FVC), and FEV_1_/FVC was 4.25% (87/2045), 12.81% (262/2045), and 1.47% (30/2045), respectively. With increasing worker’s age, the prevalence of abnormal pneumoconiosis-like changes (*p* = 0.0065), FEV_1_ (*p* = 0.0002), FVC (*p* < 0.0001), and FEV_1_/FVC (*p* = 0.0055) all increased. Factors such as age, exposure duration, enterprise size, and dust exposure concentration were associated with abnormal lung function. Conclusions: Workers exposed to occupational dust have a high rate of abnormal lung function. The government, enterprises, and individuals should pay attention to occupational dust exposure, and various effective measures should be actively taken to protect the life and health of workers.

## 1. Introduction

Pneumoconiosis is a systemic disease mainly caused by the diffuse fibrosis of lung tissues due to the long-term inhalation of dust and retention in the lungs during occupational activities. Fine dust particles are inhaled into the alveoli, thus reducing the ability of these sacs to retain oxygen and causing damage to the lungs. The initial effects of dust on the lungs are characterized by reversible respiratory symptoms and worsened lung function [1]. Occupational dust exposure can induce pulmonary inflammatory cascades and structural damage, leading to decreased lung function and other dust-related lung diseases, including sinus disease, respiratory irritation, and chronic obstructive pulmonary disease (COPD), which is globally recognized as a serious public health problem [2,3,4,5].

Occupational pneumoconiosis is one of the major diseases affecting occupational health. Whether in developed or developing countries, workers’ health is affected by occupational hazards. Pneumoconiosis is currently the most common occupational disease in China. In 2020, 14,367 new cases of occupational pneumoconiosis were recorded in China, accounting for about 84.19% of the total number of newly reported occupational disease occurrences that year [6]. The European Community Respiratory Health Survey also stated that workers exposed to biological dusts, gases, fumes, and pesticides have a high incidence of COPD, with an attributable proportion of 21% of the total population [7].

Pulmonary function parameters are reliable features for assessing airway physiology and pathology. Quantitative pulmonary function testing is an important indicator of respiratory health and clinical diagnosis of COPD and asthma [8]. Low lung function levels are a risk factor for increased morbidity and mortality [9]. Common pulmonary function evaluation indicators include forced expiratory volume in one second (FEV_1_), forced vital capacity (FVC), and the FEV_1_/FVC ratio [10]: FEV_1_ is the expiratory volume exhaled at the end of the first second of the maximal forced expiratory maneuver; FVC is the total expiratory volume from one maximal forced expiratory maneuver; and FEV_1_/FVC is the ratio of FEV_1_ to FVC, and can be used to identify obstructive or restrictive lung function impairments. Occupational dust exposure is positively correlated with decreased lung function [8,11].

Occupational pneumoconiosis has many risk factors, including occupational exposure, dust type, being underweight, length of exposure, age, smoking, and some rare genetic syndromes [7,12,13,14,15]. Using these risk factors to develop targeted and effective strategies is an effective way to protect workers’ health. In every industry, a safe workplace is critical to achieve the highest levels of productivity [16]. Primary preventive interventions are important for reducing dust exposure in the workplace, and identifying and addressing potential occupational hazards in the workplace are a major issue in ensuring workplace safety and health and are critical to eliminate the burden associated with occupational pneumoconiosis. Although companies and factories have made remarkable progress in controlling dust concentrations, a considerable number of workers are still at risk of developing lung diseases.

Respiratory problems caused by occupational dust exposure are underrepresented, and scientific evidence for such health problems in China is limited. In addition, the workers’ awareness of occupational protection is relatively weak. In view of the potential correlation of occupational dust exposure and lung function decline with the occurrence of lung diseases, this study aims to determine the occupational health of workers exposed to dust and the risk factors for lung function decline, to provide recommendations for the development of corresponding occupational disease-prevention strategies.

## 2. Materials and Methods

### 2.1. Study Subjects

**Figure 1:**
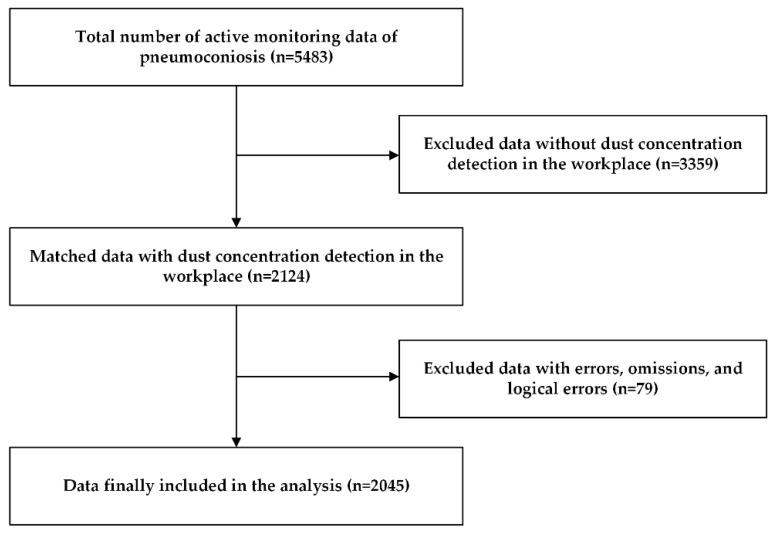
Flowchart of the enrolled study subjects.

This study surveyed a group of dust-exposed workers from the Chongqing Municipal Key Occupational Disease Surveillance Project in 2021, including 5483 active monitoring data of pneumoconiosis matching 2124 pieces of dust concentration detection data in the workplace. After data with errors, omissions, and logical errors were excluded, the data of 2045 people were finally included in the analysis (Figure 1). Workers’ occupational health examination and workplace dust concentration testing were performed by public institutions that meet relevant national requirements and regulations and were qualified to carry out occupational health inspections and workplace testing.

### 2.2. Data Collection

In this study, we asked for some information while examining the health of the workers, and at the same time detected the dust concentration in the working environment of the workers. Clinical data, including age, gender, height, weight, occupational history (including the size of the working enterprise, position, type of dust exposure, exposure start time, and exposure end time), chest X-ray test results, and pulmonary function test results (FEV_1_, FVC, and FEV_1_/FVC ratio), were obtained from the physical examination report. Workplace dust concentration was measured by professional mine dust samplers in certain locations (AKFC-92A model, ShunFa, Changshu, China). Individual dust exposure concentration was measured by matching the monitoring results of the dust concentration in the workplace according to the information of the work unit, position, and type of work.

Body mass index (BMI) was defined as weight/height^2^ and classified as underweight (<18.5 kg/m^2^), normal (18.5–24.9 kg/m^2^), and overweight/obese (≥25.0 kg/m^2^). Workplace dust concentration was defined as follows. According to China’s National Occupational Health Standard (GBZ 2.1-2019), different types of dust have different permissible concentration–time weighted average (PC-TWA) requirements [17]. Each dust has a PC-TWA limit for total dust, and some dusts have a PC-TWA limit for the respiratory dust limit. As long as one of the two limits exceeds the corresponding standard value, it can be defined as abnormal. For example, when the amount of silica dust is 10% ≤ free SiO_2_ content ≤ 50%, the PC-TWA value of the total dust is 1 mg/m^3^ and that of the respirable dust is 0.7 mg/m^3^. When the amount of silica dust is 50% ≤ free SiO_2_ content ≤ 80%, the PC-TWA value of the total dust is 0.7 mg/m^3^ and that of the respirable dust is 0.3 mg/m^3^. For limestone dust, the PC-TWA value of the total dust is 8 mg/m^3^ and that of the respirable dust is 4 mg/m^3^. In this report, the dust concentration of items on the site was defined as qualified or abnormal according to whether it exceeds the standard limit of total dust or respirable dust.

Digital radiography (DR) chest X-ray and X-ray high-kilovolt chest X-ray were used to comprehensively judge the presence of pneumoconiosis-like changes according to China’s National Occupational Health Standard (GBZ 70-2015) [18]. The Chinese “Diagnostic Criteria of Pneumoconiosis” classified pneumoconiosis as stage I, II, or III according to the size, profusion, and distribution of opacities on the chest X-ray, which is very similar to the classification system of the International Labor Organization (ILO) criteria [19]. Pulmonary function was defined as abnormal when FEV_1_ < 70%, FVC < 80%, or FEV_1_/FVC < 0.7 [20,21]. FEV_1_ and FVC were expressed using predicted percentages.

### 2.3. Statistical Analysis

Frequency and percentage were used to describe the data, and Chi-square or Fisher’ s exact tests were used for categorical data. Stepwise logistic regression was adopted for multivariate analysis. The odds ratio (OR) and 95% confidence interval (CI) were calculated [19]. Statistical significance was considered at *p* < 0.05. Statistical analysis was performed using SAS 9.4 software (SAS Institute, Cary, NC, USA).

## 3. Results

### 3.1. Demographic Characteristics

Among the 2045 workers included in the analysis, 1537 were male (75.16%). Meanwhile, 61.52% of the workers were from small enterprises, 44.30% were over 50 years old, 49.44% had a working age of less than or equal to 5 years, 62.05% had a normal BMI range, and 91.54% were exposed to silica dust (Table 1).

### 3.2. Occurrence of Pneumoconiosis-like Changes

The prevalence of pneumoconiosis-like changes was 0.83% (17/2045), and all cases were found in males. In particular, 88.24% (15/17) occurred in workers from small businesses and 82.35% (14/17) in patients older than 50 years (Table 1).

### 3.3. Occurrence of Lung Function

The prevalence of abnormal FEV_1_, FVC, and FEV_1_/FVC was 4.25% (87/2045), 12.81% (262/2045), and 1.47% (30/2045), respectively. With an increase in worker’s age, the prevalence of abnormal FEV_1_ (*p* = 0.0002), FVC (*p* < 0.0001), and FEV_1_/FVC (*p* = 0.0055) also increased. The prevalence of abnormal FEV_1_ (*p* = 0.0412) and FEV_1_/FVC (*p* = 0.0218) in workers with an abnormal dust exposure concentration was higher than that in workers with normal exposure, and the prevalence of abnormal FEV_1_ in workers from small-scale enterprises was higher than that in workers from micro- and medium-sized enterprises (*p* < 0.0001) (Table 2).

### 3.4. Multivariate Logistic Analysis

In the multivariate analysis, pneumoconiosis-like changes, FEV_1_, FVC, and FEV_1_/FVC were used as dependent variables (0 = normal; 1 = abnormal), and the dust exposure concentration, enterprise size, gender, age, exposure time, and BMI were included as independent variables. Model 1 contained all types of dust, while Model 2 contained only silica dust. The result of Model 1 showed that workers with an exposure time >10 years were more likely to have pneumoconiosis-like changes than those with an exposure time ≤5 years (OR = 3.321, 95%CI = 1.138–9.688). Compared with that in workers from micro enterprises, the prevalence of abnormal FEV_1_ was higher in workers from small enterprises (OR = 2.246, 95%CI = 1.02–4.942) and lower in workers from medium enterprises (OR = 0.151, 95%CI = 0.031–0.737). Compared with that in workers aged ≤40 years old, the prevalence of abnormal FVC was higher in workers aged >50 years (OR = 2.423, 95%CI = 1.557–3.772). Compared with that in normal workers, the prevalence of abnormal FEV_1_/FVC was higher in workers with an abnormal dust exposure concentration (OR = 2.461, 95%CI = 1.048–5.775). The result of Model 2 was similar to that of Model 1 (Table 3).

## 4. Discussion

Occupational dust exposure can lead to decreased lung function and dust-related lung disease. In this study, the association between occupational dust exposure and abnormal lung function in Chinese workers was assessed. Results showed that the prevalence of pneumoconiosis-like changes was 0.83% (17/2045), and the prevalence of abnormal FEV_1_, FVC, and FEV_1_/FVC was 4.25% (87/2045), 12.81% (262/2045), and 1.47% (30/2045), respectively. The abnormal lung function of workers is not optimistic. Relevant departments and agencies are recommended to take effective intervention measures to reduce the risks caused by workers’ exposure to dust.

The results show that enterprise size was associated with the decreased lung function of dust-exposed workers. Workers in micro and small enterprises had a high probability of developing pneumoconiosis-like changes and decreased lung function. A possible reason is that large enterprises pay attention to the occupational protection of employees and invest in occupational health education and protective facilities for employees. This result suggests that companies should pay more attention to the hazards of occupational dust exposure to workers. In addition to taking interventions to reduce the exposure concentration of dust in the working environment, such as the use of closed operating instruments and sprinkler operations in the working environment, it is also necessary to provide and supervise workers to wear protective equipment such as masks or face shields. In addition, it is necessary to improve the occupational health management system, including routine occupational health examinations and corresponding occupational health education systems.

Age is also a key influencing factor. With an increase in the worker’s age, various functions of the body exhibit varying degrees of decline. This study showed that lung function abnormalities tended to increase with age, which is similar to previous findings [22]. Whether the dust exposure concentration exceeds the standard, the duration of exposure must be determined to assess the effect of occupational dust exposure on the deterioration in lung function. Many studies showed that workers with high exposure to respirable dust had a high incidence of abnormal lung function [23,24]. In the present study, excessive dust exposure concentrations were closely related to abnormal lung function, especially FEV_1_/FVC. However, we also found that dust exposure concentration is a protective factor for FVC and a detrimental factor for FEV_1_/FVC. This may be related to the healthy worker effect. The workers who entered the study were more tolerant of abnormal FVC, while those who were not suitable for abnormal FVC may have already been transferred from their posts and did not enter the study. This may also explain why the prevalence of abnormal FVC at an abnormal dust exposure concentration was lower than that under normal exposure. We found similar results in other studies [25,26,27]. However, the current results showed no significant relationship between the duration of exposure and the decline in lung function, which is inconsistent with previous studies [2,28]. A possible reason for this difference is that similar exposure times do not necessarily imply similar total doses of exposure. Other causes include a healthy worker effect and recall bias. These results also suggest that, for workers exposed to particularly high concentrations of dust, the use of dust-proof equipment should be combined with shorter working hours or an appropriate shift system to reduce hazards [19,24].

This study revealed that factors such as age, exposure time, and dust exposure concentration are all related to abnormal lung function. This work aims to provide a basis for formulating corresponding prevention and control measures according to these high-risk factors, to reduce the occupational exposure of workers to dust. An important step in reducing exposure levels is the relevant training of workers [29,30]. Many workers do not wear protective equipment, such as dust masks, in environments with high dust concentrations, due to their limited understanding of good work habits or their own weak awareness of protection. In addition, enterprises have not been able to supervise workers in a timely manner. These reasons seriously threaten the life and health of workers.

In order to effectively protect the occupational health of workers, we can refer to the collective prevention measures based on the STOP principle. This prevention strategy combines four elements: system (S), technical (T), organizational (O), and personal (P) measures [31]. Systematic measures may include rigorous design to keep the workplace in a safe and hygienic condition, such as making workplace cleaning part of the daily routine, designing workplaces with surfaces that facilitate cleaning, and arranging equipment to facilitate air flow. Technical measures may include reducing the generation and flow of dust in the air as much as possible, such as using wet operations to reduce the generation of dust, setting up shielding covers and fences to block the flow of dust, and using air filtration and ventilation to promote the elimination of dust. Organizational measures may include the supervision and management of workers, such as regular measurement of the dust concentration in the environment, strengthening the health education of workers to promote them to form safe work habits, and regularly arranging occupational health examinations. Personal measures may focus on respiratory protection, including personal protective equipment such as masks, goggles, and gowns.

Proper ventilation, good work habits, a hygienic workplace, safe and healthy occupational training, and regular occupational health examinations are key factors in controlling respirable dust exposure and reducing occupational lung disease [32,33,34,35]. Studies have found that the use of ventilated cabs can reduce exposure to dust from engineering operations by approximately 6 times, and the use of wet operations can significantly reduce the dust exposure of workers by 3 times [32]. In addition to formulating corresponding policies and measures according to the hazards and characteristics of occupational diseases, the government’s occupational health supervision and management department should supervise and urge enterprises to implement these policies and measures. All enterprises should also pay attention to the occupational health of workers, take engineering control measures, implement health education, among other methods, to reduce the occupational dust exposure of workers, and actively organize and arrange occupational health examinations for workers to detect potential health risks as soon as possible. Workers exposed to dust should participate in studies of occupational disease prevention, to gain knowledge and protection skills, enhance their own awareness of protection, and take corresponding protection.

### Study Limitations

This study recognizes limitations that should be addressed in follow-up studies. First is the cross-sectional nature of this work. Although this study shows an association between each independent variable and the dependent variable, it cannot prove a causal relationship. Second, this study lacks an analysis of smoking data. Although some studies have questioned the etiological relationship between smoking and pneumoconiosis, with the advancement of science and technology, more and more studies have shown that smoking is a high-risk factor for decreased lung function and pneumoconiosis [4,36,37,38,39,40]. Another limitation is related to the healthy worker effect, which means that workers with respiratory symptoms or even pneumoconiosis may have left their jobs and were not included in our analysis. This may lead to an underestimation the effect of the hazard factor. The main factors that cause changes in workers’ pulmonary function are the amount of dust entering the body, the nature of the dust, and the time it acts on the body. However, this study lacks research on specific dust concentrations, contact time, and the use of personal protective equipment; so, it has certain limitations, and more in-depth research is needed.

## 5. Conclusions

This study showed that workers exposed to occupational dust have a high prevalence of abnormal lung function. Factors such as age, exposure duration, enterprise size, and dust exposure concentration were associated with abnormal lung function. Precautions must be taken to reduce the occupational exposure of dust-exposed workers. The government, enterprises, and individuals should pay attention to occupational dust exposure, and various effective measures should be actively taken to protect the life and health of workers.

## Figures and Tables

**Table 1:** Factors related to pneumoconiosis-like changes.

Characteristics	Total		Pneumoconiosis-like Changes		Chi-Square	*p*-Value
	N	%	No n (%)	Yes n (%)		
**Dust exposure concentration**					0.0539	0.8163
Normal	899	43.96%	892 (99.22%)	7 (0.78%)		
Abnormal	1146	56.04%	1136 (99.13%)	10 (0.87%)		
**Enterprise size**					-	**0.0125 ^a^**
Micro	235	11.49%	233 (99.15%)	2 (0.85%)		
Small	1258	61.52%	1243 (98.81%)	15 (1.19%)		
Medium	552	26.99%	552 (100.00%)	0 (0.00%)		
**Gender**					-	**0.0105 ^a^**
Male	1537	75.16%	1520 (98.89%)	17 (1.11%)		
Female	508	24.84%	508 (100.00%)	0 (0.00%)		
**Age**					10.0673	**0.0065**
≤40	434	21.22%	433 (99.77%)	1 (0.23%)		
40–50	705	34.47%	703 (99.72%)	2 (0.28%)		
>50	906	44.30%	892 (98.45%)	14 (1.55%)		
**Exposure time (years)**					9.0236	**0.0110**
≤5	1011	49.44%	1005 (99.41%)	6 (0.59%)		
5–10	649	31.74%	646 (99.54%)	3 (0.46%)		
>10	385	18.83%	377 (97.92%)	8 (2.08%)		
**Type of dust**					-	0.1804 ^a^
Coal dust	8	0.39%	8 (100.00%)	0 (0.00%)		
Limestone dust	45	2.20%	45 (100.00%)	0 (0.00%)		
Cement dust	120	5.87%	117 (97.50%)	3 (2.50%)		
Silica dust	1872	91.54%	1858 (99.25%)	14 (0.75%)		
**BMI (kg/m^2^)**					7.4922	**0.0236**
<18.5	36	1.76%	36 (100.00%)	0 (0.00%)		
18.5–24.9	1269	62.05%	1253 (98.74%)	16 (1.26%)		
≥25	740	36.19%	739 (99.86%)	1 (0.14%)		

BMI: body mass index. ^a^ Fisher’s exact test method.

**Table 2:** Factors related to abnormal lung function.

Characteristics	Total		FEV_1_		Chi-Square	*p*-Value	FVC		Chi-Square	*p*-Value	FEV_1_/FVC		Chi-Square	*p*-Value
	N	%	Normal (≥70%)	Abnormal (<70%)			Normal (≥80%)	Abnormal (<80%)			Normal (≥0.7)	Abnormal (<0.7)		
**Dust exposure concentration**					4.1659	**0.0412**			7.7046	**0.0055**			5.2587	**0.0218**
Normal	899	43.96%	870 (96.77%)	29 (3.23%)			763 (84.87%)	136 (15.13%)			892 (99.22%)	7 (0.78%)		
Abnormal	1146	56.04%	1088 (94.94%)	58 (5.06%)			1020 (89.01%)	126 (10.99%)			1123 (97.99%)	23 (2.01%)		
**Enterprise size**					33.162	**<0.0001**			70.1565	**<0.0001**			2.6056	0.2718
Micro	235	11.49%	228 (97.02%)	7 (2.98%)			191 (81.28%)	44 (18.72%)			234 (99.57%)	1 (0.43%)		
Small	1258	61.52%	1180 (93.80%)	78 (6.20%)			1055 (83.86%)	203 (16.14%)			1236 (98.25%)	22 (1.75%)		
Medium	552	26.99%	550 (99.64%)	2 (0.36%)			537 (97.28%)	15 (2.72%)			545 (98.73%)	7 (1.27%)		
**Gender**					1.3675	0.2422			0.5667	0.4516			2.1596	0.1417
Male	1537	75.16%	1467 (95.45%)	70 (4.55%)			1345 (87.51%)	192 (12.49%)			1511 (98.31%)	26 (1.69%)		
Female	508	24.84%	491 (96.65%)	17 (3.35%)			438 (86.22%)	70 (13.78%)			504 (99.21%)	4 (0.79%)		
**Age**					17.4964	**0.0002**			34.1521	**<0.0001**			10.3992	**0.0055**
≤40	434	21.22%	430 (99.08%)	4 (0.92%)			408 (94.01%)	26 (5.99%)			431 (99.31%)	3 (0.69%)		
40–50	705	34.47%	675 (95.74%)	30 (4.26%)			624 (88.51%)	81 (11.49%)			700 (99.29%)	5 (0.71%)		
>50	906	44.30%	853 (94.15%)	53 (5.85%)			751 (82.89%)	155 (17.11%)			884 (97.57%)	22 (2.43%)		
**Exposure time (years)**					0.4491	0.7989			4.2804	0.1176			1.5866	0.4523
≤5	1011	49.44%	967 (95.65%)	44 (4.35%)			867 (85.76%)	144 (14.24%)			995 (98.42%)	16 (1.58%)		
5–10	649	31.74%	624 (96.15%)	25 (3.85%)			579 (89.21%)	70 (10.79%)			638 (98.31%)	11 (1.69%)		
>10	385	18.83%	367 (95.32%)	18 (4.68%)			337 (87.53%)	48 (12.47%)			382 (99.22%)	3 (0.78%)		
**Type of dust**					-	**0.0209 ^a^**			18.6939	**0.0003**			-	0.4812 ^a^
Coal dust	8	0.39%	8 (100.00%)	0 (0.00%)			8 (100.00%)	0 (0.00%)			8 (100.00%)	0 (0.00%)		
Limestone dust	45	2.20%	45 (100.00%)	0 (0.00%)			44 (97.78%)	1 (2.22%)			45 (100.00%)	0 (0.00%)		
Cement dust	120	5.87%	120 (100.00%)	0 (0.00%)			117 (97.50%)	3 (2.50%)			120 (100.00%)	0 (0.00%)		
Silica dust	1872	91.54%	1785 (95.35%)	87 (4.65%)			1614 (86.22%)	258 (13.78%)			1842 (98.40%)	30 (1.60%)		
**BMI (kg/m^2^)**					3.0123	0.2218			0.5834	0.7470			0.7088	0.7016
<18.5	36	1.76%	35 (97.22%)	1 (2.78%)			31 (86.11%)	5 (13.89%)			36 (100.00%)	0 (0.00%)		
18.5–24.9	1269	62.05%	1222 (96.30%)	47 (3.70%)			1112 (87.63%)	157 (12.37%)			1249 (98.42%)	20 (1.58%)		
≥25	740	36.19%	701 (94.73%)	39 (5.27%)			640 (86.49%)	100 (13.51%)			730 (98.65%)	10 (1.35%)		

FEV_1_: forced expiratory volume in one second; FVC: forced vital capacity. ^a^ Fisher’s exact test method.

**Table 3:** Multivariate logistic regression analysis of pneumoconiosis-like changes and abnormal lung function.

Dependent Variable	Model 1 (All Types of Dust)				Model 2 (Silica Dust)			
	Independent Variable	OR	95%CI	*p*-Value	Independent Variable	OR	95%CI	*p*-Value
**Pneumoconiosis-like changes**	**Age**				**Exposure time (years)**			
	≤40	Reference			≤5	Reference		
	40–50	1.046	0.094–11.637	0.3408	5–10	1.136	0.253–5.095	0.3385
	>50	5.838	0.761–44.809	**0.0094**	>15	4.558	1.326–15.668	**0.0072**
	**Exposure time (years)**							
	≤5	Reference						
	5–10	0.776	0.193–3.119	0.1825				
	>15	3.321	1.138–9.688	**0.0087**				
**FEV_1_**	**Enterprise size**				**Enterprise size**			
	Micro	Reference			Micro	Reference		
	Small	2.246	1.020–4.942	**<0.0001**	Small	2.496	1.134–5.495	**<0.0001**
	Medium	0.151	0.031–0.737	**0.0019**	Medium	0.161	0.033–0.787	**0.0020**
	**Age**				**Age**			
	≤40	Reference			≤40	Reference		
	40–50	3.951	1.376–11.344	0.0682	40–50	3.674	1.277–10.57	0.0848
	>50	4.813	1.722–13.457	**0.0038**	>50	4.429	1.581–12.404	**0.0062**
**FVC**	**Dust exposure concentration**				**Dust exposure concentration**			
	Normal	Reference			Normal	Reference		
	Abnormal	0.676	0.517–0.884	**0.0043**	Abnormal	0.690	0.526–0.907	**0.0077**
	**Enterprise size**				**Enterprise size**			
	Micro	Reference			Micro	Reference		
	Small	0.932	0.646–1.345	**<0.0001**	Small	1.028	0.712–1.485	**<0.0001**
	Medium	0.157	0.084–0.291	**<0.0001**	Medium	0.158	0.084–0.297	**<0.0001**
	**Age**				**Age**			
	≤40	Reference			≤40	Reference		
	40–50	1.767	1.107–2.819	0.4411	40–50	1.722	1.068–2.775	0.4313
	>50	2.423	1.557–3.772	**<0.0001**	>50	2.279	1.448–3.586	**0.0003**
**FEV_1_/FVC**	**Dust exposure concentration**				**Dust exposure concentration**			
	Normal	Reference			Normal	Reference		
	Abnormal	2.461	1.048–5.775	**0.0386**	Abnormal	2.441	1.040–5.730	**0.0404**
	**Age**				**Age**			
	≤40	Reference			≤40	Reference		
	40–50	0.974	0.231–4.104	0.2508	40–50	0.939	0.223–3.955	0.2400
	>50	3.323	0.987–11.189	**0.0044**	>50	3.175	0.943–10.694	**0.0054**

## Data Availability

The datasets involved in the current study are not publicly available due to privacy but are available from the author Wei He on reasonable request.
